# The global health burden of pediatric chronic kidney disease: An analysis of the Global Burden of Disease database from 1990 to 2021

**DOI:** 10.1371/journal.pone.0323257

**Published:** 2025-05-27

**Authors:** Manan Raina, Raghav Shah, Jieji Hu, Bryce Pember, Thomas Cahill, Timothy Bunchman, Hui Kim Yap, Mignon McCulloch

**Affiliations:** 1 Hawken School, Chesterland, Ohio, United States of America; 2 College of Medicine, Northeast Ohio Medical University, Rootstown, Ohio, United States of America; 3 Pediatric Nephrology and Transplantation, Children’s Hospital of Richmond, Virginia Commonwealth University, Richmond, Virginia, United States of America; 4 Department of Pediatrics, Yong Loo Lin School of Medicine, National University of Singapore, Singapore, Singapore; 5 Khoo Teck Puat-National University Children’s Medical Institute, National University Hospital, Singapore, Singapore; 6 Department of Paediatric Nephrology, Red Cross Children’s Hospital, University of Cape Town, Rondebosch, Cape Town, South Africa; HERD International, NEPAL

## Abstract

**Background:**

Chronic kidney disease (CKD) poses a significant global health challenge; however, its burden on pediatric populations remains underexplored. This study assesses the incidence, prevalence, mortality, and disability-adjusted life years (DALYs) of pediatric CKD attributable to type 1 diabetes mellitus, type 2 diabetes mellitus, hypertension, glomerulonephritis, and congenital anomalies of the kidney and urinary tract from 1990 to 2021.

**Methods:**

Data were extracted from the Global Burden of Disease (GBD) 2021 database. CKD burden was stratified by etiology, age, sex, sociodemographic index (SDI), and geography. Average annual percentage changes (AAPCs) in incidence, prevalence, mortality, and DALYs were calculated.

**Results:**

Globally, CKD-related mortality and DALYs decreased for type 1 diabetes mellitus (-2.176% AAPC), type 2 diabetes mellitus (-1.556%), glomerulonephritis (-0.854%), hypertension (-0.800%), and congenital anomalies of the kidney and urinary tract (-2.143%). The incidence of hypertension incidence increased (+1.299%). Boys experienced higher incidence and prevalence rates for all etiologies, while girls had more significant reductions in mortality and DALYs. High-SDI regions showed the steepest declines in CKD burden, while low-SDI regions experienced limited reductions and increasing hypertension prevalence.

**Conclusions:**

Pediatric CKD burden reflects disparities by etiology, geography, and SDI. Interventions to improve early diagnosis, healthcare access, and targeted management strategies, particularly in low-SDI regions, are essential. Addressing obesity and improving treatment for glomerulonephritis are priorities. Standardized diagnostic criteria and broader global efforts are needed to mitigate the burden of pediatric CKD.

## Introduction

Chronic kidney disease (CKD) is one of the most prevalent non-communicable health concerns, affecting over 10% of the global population, amounting to more than 850 million individuals [1,2]. CKD is the third fastest-growing cause of death worldwide and the only non-communicable disease to exhibit a continued rise in age-adjusted mortality; by 2040, CKD is projected to be the 5th highest cause of years of life lost in the world [3,4]. While studies show that CKD is more likely in older populations, data is scarce in the pediatric population and the true epidemiology and global health burden in children needs to be clarified [5]. Implications for pediatric CKD also include its effect on growth and the impact on the child’s adult life, considerations not present in the adult population [6]. Pediatric CKD is most often asymptomatic initially, making it difficult to obtain reliable data on the incidence and prevalence of the early stages of CKD [7]. Based on the current kidney registries and epidemiological studies, the prevalence of pediatric CKD is approximately 30–100 cases per million age-related population, suggesting that about 1 in every 10,000 children has CKD [7]. This number may be underreported as well, with reported cases in resource-limited countries likely confined to patients at tertiary care centers, or going unreported all together [8].

The latest 2024 Kidney Disease: Improving Global Outcomes (KDIGO) CKD guidelines have further expanded on the assessment and testing for CKD in children, which should continue to standardize the pediatric CKD definition [9]. CKD is defined as abnormalities of kidney structure or function for more than 3 months, with the exception of neonates who have obvious congenital kidney disease [9]. Children with CKD are at an increased risk of chronic cardiovascular disease, obstructive respiratory disease, and mental illness in adulthood [10]. Thus, timely diagnosis, intervention, and monitoring are of utmost importance in the pediatric population, which requires an understanding of the etiologies behind CKD in children. Pediatric CKD is primarily caused by congenital anomalies of the urinary tract (CAKUT), but can also be caused by type 1 diabetes mellitus (T1DM), type 2 diabetes mellitus (T2DM), hypertension, and glomerulonephritis [11].

Understanding the impacts of pediatric CKD caused by these etiologies is crucial for developing effective treatment and management strategies. The pediatric literature currently does not have a comparative study on the health burden of CKD due to different etiologies. To the best of our knowledge, this is the first study conducted on the incidence, prevalence, mortality, and DALYs of pediatric CKD etiologies on a national, regional, and global scale.

### Objectives

This study utilizes the Global Burden of Diseases (GBD) database and data on incidence, prevalence, mortality, and DALYs to provide an updated estimate of the global health burden of pediatric CKD etiologies (T1DM, T2DM, hypertension, glomerulonephritis, and CAKUT) in different geographic locations, sociodemographic indexes, ages, and sexes. This study also provides a comparative analysis to delineate the health burden of pediatric CKD from 1990–2021. We utilized the Strengthening the Reporting of Observational Studies in Epidemiology (STROBE) reporting guidelines.

## Materials and methods

### Data source

Data on the global burden of pediatric CKD attributable to hypertension, T1DM, T2DM, and glomerulonephritis were obtained from GBD 2021 (accessed August 5^th^, 2024) (S1 File). The GBD database did not include CKD attributable to CAKUT; therefore, we included urological congenital abnormalities from the database. The GBD database utilized data primarily from end-stage kidney registries that included CKD etiologies. Individual patient data was not available. The GBD data for CKD due to overall DM were more widely available than data by type of DM. As such, the GBD data for CKD due to overall DM, T1DM, and T2DM was modeled through Bayesian meta-regression (DisMod- MR 2.1) with diabetes prevalence and mean systolic blood pressure as country-level covariates to obtain estimates of each by location, year, age, and sex [12]. The proportions of CKD due to T1DM and T2DM were then scaled to sum to the proportion of overall DM by location, year, age, and sex. The incidence, prevalence, mortality, and DALYs of each pediatric CKD etiology were obtained and further categorized by age, sex, sociodemographic index (SDI), and region. Since this analysis was performed using publicly available de-identified patient data, IRB approval was not required.

### Study population

The study population included children aged 19 years and under. The GBD defined CKD as a chronic, progressive condition of the kidney, lasting 3 months or more, with a loss in its key function to filtrate blood to produce urine. The GBD used the CKD-Epi eGFR equation for those 18 years or older and used the Schwartz equation for those younger than 18.

### DALYs

Disability-adjusted life years (DALYs) were calculated as the sum of years of life lost and years lived with disability, calculated per GBD location. Years of life lost are years of life lost due to premature mortality and years lived with disability are years of life lived with any short-term or long-term health loss.

### APC and AAPC

The average annual percentage change (AAPC) over 1990–2021 for these measures at the global and regional levels has been reported. Further, the global AAPC has been stratified by age, sex, and socio-demographic index (SDI) for each etiology of pediatric CKD.

### SDI

The sociodemographic index (SDI) developed by the Global Burden of Disease Center is a composite score strongly associated with global health outcomes. Specifically, it is a mean index number of total fertility rate under the age of 25, mean education for those ages 15 and older, and lag distributed income per capita, categorized into quintiles [13]. Several studies have correlated SDI with mortality, life expectancy, and healthy life expectancy, among other variables closely related to population health [14]. We utilized the AAPC of the SDI to represent an appropriate point of chronologic interpretation. The SDI for the 204 countries and territories included in the GBD database were categorized into low, low-middle, middle, middle-high, and high.

### Uncertainty intervals

In GBD 2021, every estimate was calculated 500 times, each time sampling from distributions rather than point estimates for data inputs, data transformations, and model choice [15]. The 95th uncertainty interval is determined by the 25th and 975th value of the 1,000 values after ordering them from smallest to largest.

### Statistical analysis

The incidence, prevalence, death, and DALYs rates per 100,000 children and adolescents have been reported for CKD due to four diseases a) T1DM; b) T2DM; c) hypertension; and d) glomerulonephritis. To estimate CAKUT, we included the incidence, prevalence, death, and DALYs rate for congenital urological abnormalities. The AAPC over 1990–2021 for these measures at the global and regional levels have been reported. Further, the global AAPC has been stratified by age, sex, and SDI for CKD due to the five diseases. The SPSS and R statistical packages were utilized.

## Results

### Incidence

Globally, the overall incidence rates per 100,000 children and adolescents between 1990 and 2021 showed a decreasing trend for CKD due to T2DM [AAPC -0.591% (95% CI -0.837% - 0.075%)], CKD due to glomerulonephritis [-0.077% (-0.111% - -0.004%)], CKD due to T1DM [-0.058% (-0.090% - -0.004%)], and CAKUT [-1.414% (-3.160 - 0.286%) but an increasing trend for CKD due to hypertension [1.299% (0.863% - 1.835%)] (**Table 1**).

**Table 1:** Average annual percentage change (AAPC) of global CKD incidence due to different etiologies in children and adolescents between 1990 and 2021.

	AAPC		95% CI
CKD due to Type 1 diabetes	−0.058%		−0.090%–−0.004%
CKD due to Type 2 diabetes	−0.591%		−0.837%–0.075%
CKD due to Hypertension	1.299%		0.863%–1.835%
CKD due to Glomerulonephritis	−0.077%		−0.111%–−0.004%
CAKUT	−1.414%		−3.160–0.286%
b. **AAPC of global CKD incidence stratified by sex.**			
	**Sex**	**AAPC**	**95% CI**
CKD due to Type 1 diabetes	Boy	−0.020%	−0.030%–0.043%
	Girl	−0.116%	−0.144%–−0.072%
CKD due to Type 2 diabetes	Boy	−0.090%	−0.395%–0.526%
	Girl	−1.282%	−1.481%–−0.855%
CKD due to Hypertension	Boy	1.673%	1.230%–2.295%
	Girl	0.801%	0.587%–1.157%
CKD due to Glomerulonephritis	Boy	−0.025%	−0.055%–0.023%
	Girl	−0.179%	−0.232%–−0.102%
CAKUT	Boy	−1.368%	−3.056%–0.421%
	Girl	−1.533%	−3.286%–0.187%
c. **AAPC of global CKD incidence stratified by age groups [wherever data available].**			
	**Age groups**	**AAPC**	**95% CI**
CKD due to Type 1 diabetes	<1 year	0.150%	0.148%–0.155%
	2-4 years	−0.394%	−0.453%–−0.346%
	<5 years	−0.210%	−0.248%–−0.186%
	5-9 years	0.323%	0.225%–0.435%
	10-19 years	1.673%	1.362%–2.050%
CKD due to Type 2 diabetes	10-19 years	−0.741%	−0.987%–−0.077%
CKD due to Hypertension	10-19 years	1.146%	0.710%–1.680%
CKD due to Glomerulonephritis	<1 year	0.121%	0.068%–0.155%
	2-4 years	−0.398%	−0.414%–−0.372%
	<5 years	−0.229%	−0.358%–−0.194%
	5-9 years	0.339%	0.196%–0.540%
	10-19 years	1.240%	0.888%–1.657%
CAKUT	<1 year	−0.176%	−1.941%–1.546%
	<5 years	−0.388%	−2.150%–1.330%
d. **AAPC of global CKD incidence stratified by socio-demographic index (SDI) index.**			
	**SDI**	**AAPC**	**95% CI**
CKD due to Type 1 diabetes	High SDI	−0.117%	−0.199%–−0.055%
	High-middle SDI	−0.349%	−0.451%–−0.134%
	Middle SDI	−0.082%	−0.213%–−0.021%
	Low-middle SDI	0.026%	0.010%–0.098%
	Low SDI	−0.109%	−0.233%–0.014%
CKD due to Type 2 diabetes	High SDI	−1.289%	−1.506%–−0.950%
	High-middle SDI	−2.491%	−2.580%–−2.206%
	Middle SDI	−0.621%	−0.865%–0.022%
	Low-middle SDI	−0.117%	−0.391%–0.252%
	Low SDI	−0.438%	−0.599%–−0.140%
CKD due to Hypertension	High SDI	0.044%	−0.266%–0.480%
	High-middle SDI	0.330%	0.187%–0.755%
	Middle SDI	1.387%	0.945%–2.042%
	Low-middle SDI	1.680%	1.428%–2.281%
	Low SDI	1.318%	1.083%–1.795%
CKD due to Glomerulonephritis	High SDI	−0.172%	−0.205%–−0.128%
	High-middle SDI	−0.522%	−0.555%–−0.480%
	Middle SDI	−0.100%	−0.181%–−0.040%
	Low-middle SDI	0.045%	0.016%–0.064%
	Low SDI	−0.108%	−0.167%–−0.071%
CAKUT	High SDI	−1.497%	−3.039%–0.038%
	High-middle SDI	−2.377%	−4.043%–−0.690%
	Middle SDI	−2.039%	−3.796%–−0.310%
	Low-middle SDI	−1.661%	−3.440%–0.126%
	Low SDI	−1.293%	−3.102%–0.473%
e. **AAPC of global CKD incidence across various regions.**			
	**Region**	**AAPC**	**95% CI**
CKD due to Type 1 diabetes	Asia	−0.252%	−0.410%–−0.151%
	Africa	−0.026%	−0.066%–0.013%
	America	0.155%	0.029%–0.282%
	Europe	−0.090%	−0.105%–0.093%
CKD due to Type 2 diabetes	Asia	−0.563%	−0.762%–0.069%
	Africa	−0.630%	−0.771%–−0.135%
	America	0.341%	−0.058%–1.155%
	Europe	−3.990%	−4.528%–−3.487%
CKD due to Hypertension	Asia	1.378%	1.065%–1.878%
	Africa	1.143%	0.894%–1.682%
	America	1.665%	1.210%–2.617%
	Europe	0.500%	0.421%–0.736%
CKD due to Glomerulonephritis	Asia	−0.245%	−0.275%–−0.202%
	Africa	−0.034%	−0.045%–−0.029%
	America	0.186%	0.123%–0.312%
	Europe	−0.378%	−0.490%–−0.294%
CAKUT	Asia	−1.942%	−3.745%–−0.164%
	Africa	−1.291%	−3.081%–0.486%
	America	−1.579%	−3.248%–0.150%
	Europe	−1.525%	−3.079%–−0.017%

**Fig 1:**
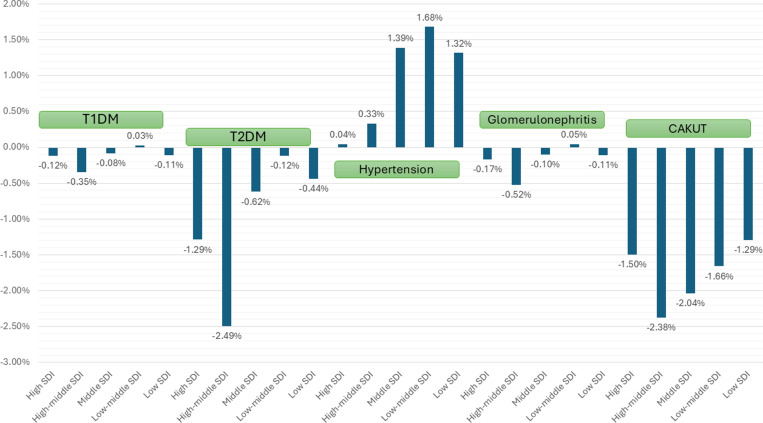
AAPC of CKD incidence due to different causes between 1990 and 2021 stratified by socio-demographic index (SDI) index.

**Fig 2:**
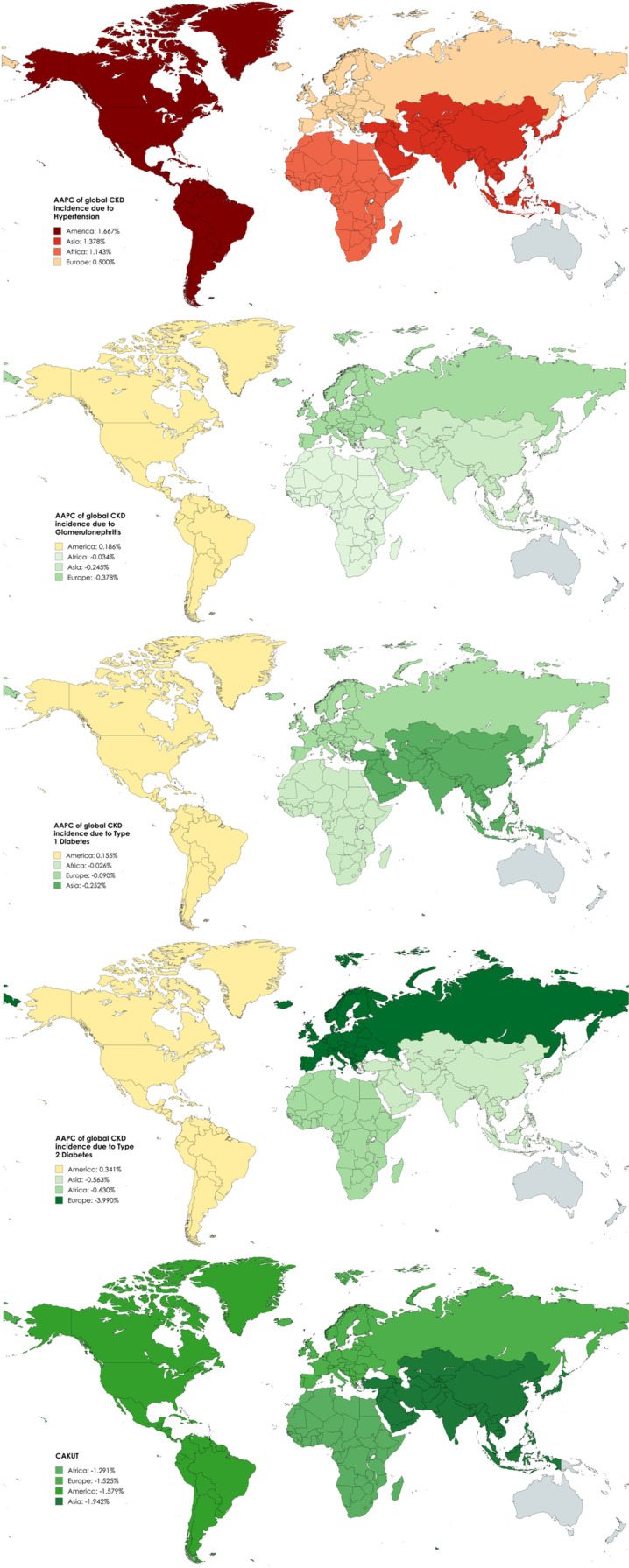
AAPC of CKD incidence due to different causes between 1990 and 2021 stratified by region.

The decrease in overall incidence over 1990–2021 for CAKUT and CKD due to T1DM, T2DM, and glomerulonephritis was observed to be higher in girls as compared with boys; however, the increase in overall incidence for CKD due to hypertension was higher in boys as compared with girls (Table 1b). Regarding variation with age, the overall incidence over 1990–2021 for CKD due to T1DM and glomerulonephritis increased in children aged >5 years and <1 years but decreased in children aged between 2 to <5 years (age-wise data was available only for T1DM and glomerulonephritis; Table 1c). With respect to the SDI, the decrease in overall incidence over 1990–2021 for CKD due to T1DM, T2DM, and glomerulonephritis was greater among those with higher SDI as compared with lower SDI (Table 1d and Figs 1 and 2). With respect to the geography, the overall incidence over 1990–2021 for CKD due to T1DM, T2DM, and glomerulonephritis decreased in Asia, Africa, and Europe but increased in America (Table 1e). The incidence of CAKUT decreased over 1990–2021 primarily in middle to high-middle SDI countries and in all four regions.

### Prevalence

Globally, the overall prevalence rates per 100,000 children and adolescents between 1990 and 2021 showed a decreasing trend for CAKUT [-0.614% (-1.989% - 0.762%) and CKD due to T2DM [AAPC -1.300% (-1.340% - -1.234%)], glomerulonephritis [-0.356% (-0.411% - -0.298%)], and hypertension [-0.066% (-0.114% - -0.021%)], but an increasing trend for T1DM [0.852% (0.762% - 0.901%)] (**Table 2**).

**Table 2:** AAPC of global CKD prevalence due to different etiologies between 1990 and 2021.

		AAPC	95% CI
CKD due to Type 1 diabetes		0.852%	0.762%–0.901%
CKD due to Type 2 diabetes		−1.300%	−1.340%–−1.234%
CKD due to Hypertension		−0.066%	−0.114%–−0.021%
CKD due to Glomerulonephritis		−0.356%	−0.411%–−0.298%
CAKUT		−0.614%	−1.989%–0.762%
b. **AAPC of global CKD prevalence stratified by sex.**			
	**Sex**	**AAPC**	**95% CI**
CKD due to Type 1 diabetes	Boy	0.715%	0.676%–0.796%
	Girl	0.972%	0.883%–1.038%
CKD due to Type 2 diabetes	Boy	−1.185%	−1.252%–−1.108%
	Girl	−1.451%	−1.485%–−1.403%
CKD due to Hypertension	Boy	0.007%	−0.030%–0.063%
	Girl	−0.161%	−0.215%–−0.113%
CKD due to Glomerulonephritis	Boy	−0.346%	−0.390%–−0.301%
	Girl	−0.387%	−0.451%–−0.340%
CAKUT	Boy	−0.501%	−1.934%–0.933%
	Girl	−0.711%	−2.040%–0.631%
c. **AAPC of global CKD prevalence stratified by age groups [wherever data available].**			
	**Age groups**	**AAPC**	**95% CI**
CKD due to Type 1 diabetes	<1 year	−1.136%	−1.194%–−1.118%
	2-4 years	−0.942%	−1.002%–−0.815%
	<5 years	−0.889%	−0.953%–−0.772%
	5-9 years	−0.555%	−0.657%–−0.377%
	10-19 years	0.933%	0.835%–0.954%
CKD due to Type 2 diabetes	10-19 years	−1.449%	−1.489%–−1.384%
CKD due to Hypertension	10-19 years	−0.218%	−0.266%–−0.172%
CKD due to Glomerulonephritis	<1 year	−0.100%	−0.315%–0.011%
	2-4 years	−0.671%	−0.749%–−0.571%
	<5 years	−0.550%	−0.639%–−0.459%
	5-9 years	−0.758%	−0.850%–−0.671%
	10-19 years	−0.372%	−0.458%–−0.316%
CAKUT	<1 year	−0.138%	−1.706%–1.478%
	<5 years	−0.150%	−1.602%–1.358%
d. **AAPC of global CKD prevalence stratified by socio-demographic index (SDI) index.**			
	**SDI**	**AAPC**	**95% CI**
CKD due to Type 1 diabetes	High SDI	0.684%	0.549%–0.708%
	High-middle SDI	1.256%	1.203%–1.349%
	Middle SDI	0.976%	0.937%–1.033%
	Low-middle SDI	0.865%	0.852%–0.872%
	Low SDI	0.749%	0.725%–0.786%
CKD due to Type 2 diabetes	High SDI	−1.519%	−1.543%–−1.424%
	High-middle SDI	−2.269%	−2.285%–−2.141%
	Middle SDI	−1.368%	−1.392%–−1.327%
	Low-middle SDI	−1.001%	−1.084%–−0.927%
	Low SDI	−1.114%	−1.194%–−0.990%
CKD due to Hypertension	High SDI	−0.200%	−0.232%–−0.175%
	High-middle SDI	−0.910%	−0.946%–−0.856%
	Middle SDI	−0.546%	−0.577%–−0.537%
	Low-middle SDI	−0.016%	−0.051%–0.032%
	Low SDI	−0.144%	−0.172%–−0.105%
CKD due to Glomerulonephritis	High SDI	−0.113%	−0.148%–−0.077%
	High-middle SDI	−0.854%	−0.920%–−0.79%
	Middle SDI	−0.301%	−0.303%–−0.296%
	Low-middle SDI	0.471%	0.469%–0.558%
	Low SDI	0.336%	0.304%–0.355%
CAKUT	High SDI	−0.768%	−1.964%–0.427%
	High-middle SDI	−1.277%	−2.542%–0.014
	Middle SDI	−0.937%	−2.289%–0.420%
	Low-middle SDI	−0.751%	−2.223%–0.771%
	Low SDI	−0.479%	−1.931%–1.009%
e. **AAPC of global CKD prevalence across various regions.**			
	**Region**	**AAPC**	**95% CI**
CKD due to Type 1 diabetes	Asia	1.017%	0.868%–1.150%
	Africa	0.602%	0.547%–0.614%
	America	0.725%	0.668%–0.781%
	Europe	1.615%	1.554%–1.679%
CKD due to Type 2 diabetes	Asia	−1.050%	−1.131%–−0.967%
	Africa	−1.296%	−1.356%–−1.263%
	America	−0.742%	−0.796%–−0.683%
	Europe	−4.000%	−4.654%–−3.603%
CKD due to Hypertension	Asia	−0.014%	−0.095%–0.085%
	Africa	0.156%	0.082%–0.182%
	America	0.234%	0.200%–0.276%
	Europe	−0.664%	−0.744%–−0.518%
CKD due to Glomerulonephritis	Asia	−0.322%	−0.417%–−0.276%
	Africa	−0.224%	−0.241%–−0.200%
	America	−0.092%	−0.111%–−0.089%
	Europe	−0.716%	−0.773%–−0.626%
CAKUT	Asia	−0.905%	−2.332%–0.548%
	Africa	−0.457%	−1.892%–0.992%
	America	−0.821%	−2.003%–0.370%
	Europe	−0.892%	−2.133%–0.381%

**Fig 3:**
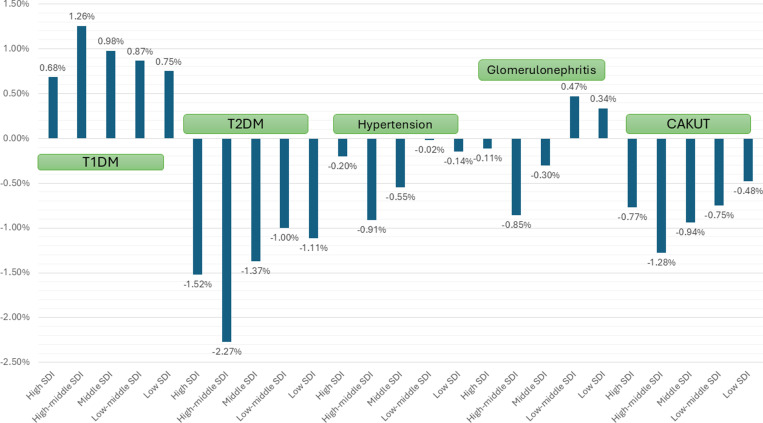
AAPC of CKD prevalence due to different causes between 1990 and 2021 stratified by socio-demographic index (SDI) index.

The decrease in overall prevalence over 1990–2021 for CAKUT and CKD due to T2DM, hypertension, and glomerulonephritis was observed to be greater in girls as compared with boys; however, the increase in overall prevalence for T1DM was higher in girls as compared with boys (Table 2b). Regarding variation with age, the overall prevalence over 1990–2021 for T1DM decreased for children aged <10 years with a declining trend but increased in children aged 10–19 years; however, for glomerulonephritis, the decline was greater in children 2–9 years as compared to those aged <1 years and 10–19 years (age-wise data was available only for T1DM and glomerulonephritis; Table 2c). With respect to the SDI, the decrease in overall prevalence over 1990–2021 for CKD due to T2DM, glomerulonephritis, and hypertension was greater among those with higher SDI as compared with lower SDI (Table 2d and Fig 3). With respect to the geography, the decrease in overall prevalence over 1990–2021 for T2DM, hypertension, and glomerulonephritis was observed to be greater in Europe as compared with Asia, America, or Africa (Table 2e). The prevalence of CAKUT decreased over 1990–2021 primarily in middle to high-middle SDI countries and in all four regions.

### Death

Globally, the overall death rates per 100,000 children and adolescents showed a decreasing trend between 1990 and 2021 for CAKUT [-2.143% (-4.345% - 0.448%)], CKD due to T1DM [-2.176% (-2.364% - -2.081%)], T2DM [-1.556% (-1.644% - -1.580%)], glomerulonephritis [-0.854% (-0.866% - -0.754%)], and hypertension [-0.800% (-0.842% - -0.778%)] (**Table 3**).

**Table 3:** AAPC of global CKD death rates due to different etiologies between 1990 and 2021.

		AAPC	95% CI
CKD due to Type 1 diabetes		−2.176%	−2.364%–−2.081%
CKD due to Type 2 diabetes		−1.556%	−1.644%–−1.580%
CKD due to Hypertension		−0.800%	−0.842%–−0.778%
CKD due to Glomerulonephritis		−0.854%	−0.866%–−0.754%
CAKUT		−2.143%	−4.345%–0.448%
b. **AAPC of global CKD death rates stratified by sex.**			
	**Sex**	**AAPC**	**95% CI**
CKD due to Type 1 diabetes	Boy	−1.942%	−1.986%–−1.886%
	Girl	−2.476%	−2.680%–−2.367%
CKD due to Type 2 diabetes	Boy	−1.270%	−1.457%–−1.200%
	Girl	−1.909%	−1.970%–−1.935%
CKD due to Hypertension	Boy	−0.568%	−0.574%–−0.519%
	Girl	−1.125%	−1.194%–−1.053%
CKD due to Glomerulonephritis	Boy	−0.693%	−0.794%–−0.472%
	Girl	−1.086%	−1.151%–−1.029%
CAKUT	Boy	−1.584%	−4.421%–1.764%
	Girl	−2.550%	−6.199%–1.422%
c. **AAPC of global CKD death rate stratified by age groups [wherever data available].**			
	**Age groups**	**AAPC**	**95% CI**
CKD due to Type 1 diabetes	<1 year	−3.227%	−3.416%–−3.274%
	2-4 years	−3.921%	−3.998%–−3.702%
	<5 years	−3.680%	−3.718%–−3.677%
	5-9 years	−2.746%	−2.784%–−2.652%
	10-19 years	−1.887%	−2.044%–−1.829%
CKD due to Type 2 diabetes	10-19 years	−1.705%	−1.793%–−1.729%
CKD due to Hypertension	10-19 years	−0.951%	−0.998%–−0.923%
CKD due to Glomerulonephritis	<1 year	−1.667%	−1.729%–−1.576%
	2-4 years	−2.229%	−2.248%–−1.879%
	<5 years	−2.025%	−2.092%–−1.929%
	5-9 years	−1.241%	−1.313%–−1.073%
	10-19 years	0.159%	0.115%–0.269%
CAKUT	<1 year	−0.892%	−3.572%–1.878%
	<5 years	−1.166%	−3.814%–1.483%
d. **AAPC of global CKD death rates AAPC of global CKD death rates.**			
	**SDI**	**AAPC**	**95% CI**
CKD due to Type 1 diabetes	High SDI	−3.193%	−3.390%–−3.086%
	High-middle SDI	−3.035%	−3.063%–−2.984%
	Middle SDI	−2.231%	−2.359%–−2.176%
	Low-middle SDI	−1.283%	−1.424%–−1.133%
	Low SDI	−1.405%	−1.642%–−1.310%
CKD due to Type 2 diabetes	High SDI	−2.587%	−2.637%–−2.434%
	High-middle SDI	−2.645%	−2.851%–−2.550%
	Middle SDI	−1.755%	−1.862%–−1.660%
	Low-middle SDI	−0.226%	−0.267%–−0.064%
	Low SDI	−0.627%	−0.865%–−0.501%
CKD due to Hypertension	High SDI	−2.055%	−2.100%–−1.925%
	High-middle SDI	−1.792%	−1.838%–−1.729%
	Middle SDI	−0.741%	−0.849%–−0.709%
	Low-middle SDI	−0.271%	−0.282%–−0.246%
	Low SDI	−0.235%	−0.333%–−0.086%
CKD due to Glomerulonephritis	High SDI	−2.177%	−2.247%–−2.070%
	High-middle SDI	−2.718%	−2.776%–−2.622%
	Middle SDI	−1.655%	−1.728%–−1.533%
	Low-middle SDI	−1.074%	−1.163%–−0.729%
	Low SDI	−1.282%	−1.309%–−1.199%
CAKUT	High SDI	−2.699%	−5.097%–−1.259%
	High-middle SDI	−3.616%	−5.605%–1.820%
	Middle SDI	−2.143%	−4.240%–−0.625%
	Low-middle SDI	−2.143%	−5.445%–1.404%
	Low SDI	−1.479%	−5.682%–2.476%
e. **AAPC of global CKD death rates across various regions.**			
	**Region**	**AAPC**	**95% CI**
CKD due to Type 1 diabetes	Asia	−2.327%	−2.446%–−2.262%
	Africa	−1.103%	−1.331%–−0.933%
	America	−1.259%	−1.524%–−1.158%
	Europe	−2.445%	−2.597%–−2.371%
CKD due to Type 2 diabetes	Asia	−1.831%	−1.862%–−1.814%
	Africa	−0.362%	−0.605%–−0.263%
	America	−0.018%	−0.095%–−0.009%
	Europe	−1.357%	−1.414%–−1.335%
CKD due to Hypertension	Asia	−0.891%	−1.004%–−0.803%
	Africa	0.094%	0.059%–0.279%
	America	−0.018%	−0.187%–0.072%
	Europe	−1.991%	−2.045%–−1.854%
CKD due to Glomerulonephritis	Asia	−1.949%	−1.991%–−1.620%
	Africa	−1.024%	−1.114%–−0.904%
	America	−1.663%	−1.847%–−1.467%
	Europe	−2.770%	−2.870%–−2.691%
CAKUT	Asia	−1.945%	−5.320%–1.156%
	Africa	−2.143%	−6.450%–2.094%
	America	−1.403%	−4.068%–0.179%
	Europe	−4.240%	−6.292%–1.584%

**Fig 4:**
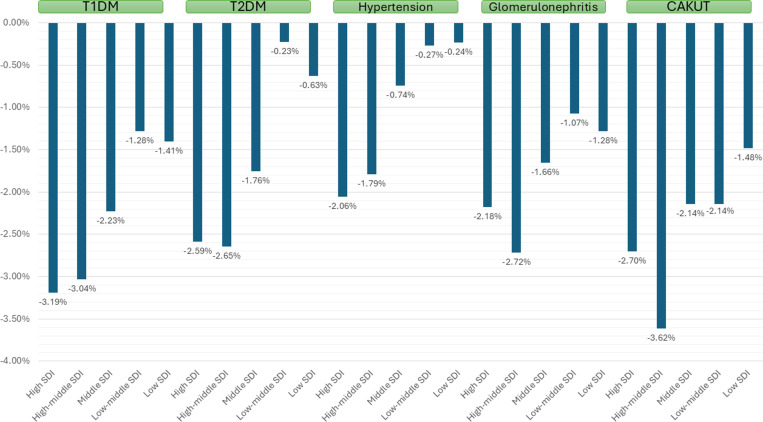
AAPC of CKD death due to different causes between 1990 and 2021 stratified by socio-demographic index (SDI) index.

The decrease in overall death over 1990–2021 was observed to be higher in girls as compared with boys (Table 3b) and in younger versus older age (age-wise data was available only for T1DM and glomerulonephritis; Table 3c). With respect to the SDI, the decrease in overall death over 1990–2021 was greater among those in high SDI as compared with low SDI with a declining trend (Table 3d and Fig 4). With respect to the geography, the decrease in overall death over 1990–2021 was observed to be higher in Europe and Asia as compared with America or Africa (Table 3e). High middle SDI countries and Europe had the highest decrease in death rates due to CAKUT.

### DALYs

Globally, the overall DALY rates per 100,000 children and adolescents showed a decreasing trend between 1990 and 2021 for CAKUT [-1.733 (-3.857% – 0.288%), CKD due to T1DM [AAPC -2.144% (-2.333% - -2.092%)], T2DM [-2.046% (-2.096% - -1.954%)], glomerulonephritis [-0.920% (-0.969% - -0.890%)], and hypertension [-0.690% (-0.698% - -0.678%)] (**Table 4**).

**Table 4:** AAPC of global CKD DALYs due to different etiologies between 1990 and 2021.

		AAPC		95% CI
CKD due to Type 1 diabetes		−2.144%		−2.333%–−2.092%
CKD due to Type 2 diabetes		−2.046%		−2.096%–−1.954%
CKD due to Hypertension		−0.690%		−0.698%–−0.678%
CKD due to Glomerulonephritis		−0.920%		−0.969%–−0.890%
CAKUT		−1.733%		−3.857–0.288%
b. **AAPC of global CKD DALYs stratified by sex.**				
	**Sex**		**AAPC**	**95% CI**
CKD due to Type 1 diabetes	Boy		−1.926%	−1.946%–−1.898%
	Girl		−2.427%	−2.655%–−2.392%
CKD due to Type 2 diabetes	Boy		−1.827%	−1.896%–−1.702%
	Girl		−2.300%	−2.358%–−2.272%
CKD due to Hypertension	Boy		−0.498%	−0.499%–−0.494%
	Girl		−0.952%	−0.962%–−0.928%
CKD due to Glomerulonephritis	Boy		−0.773%	−0.805%–−0.497%
	Girl		−1.133%	−1.148%–−1.081%
CAKUT	Boy		−1.394%	−3.930%–1.426%
	Girl		−2.191%	−4.973%–0.871%
c. **AAPC of global CKD DALYs stratified by age groups [wherever data available].**				
	**Age groups**		**AAPC**	**95% CI**
CKD due to Type 1 diabetes	<1 year		−3.216%	−3.395%–−3.168%
	2-4 years		−3.784%	−3.841%–−3.436%
	<5 years		−3.607%	−3.676%–−3.504%
	5-9 years		−2.577%	−2.666%–−2.399%
	10-19 years		−1.812%	−1.942%–−1.788%
CKD due to Type 2 diabetes	10-19 years		−2.194%	−2.199%–−2.103%
CKD due to Hypertension	10-19 years		−0.841%	−0.848%–−0.828%
CKD due to Glomerulonephritis	<1 year		−1.663%	−1.673%–−1.624%
	2-4 years		−2.171%	−2.197%–−1.801%
	<5 years		−1.993%	−1.998%–−1.876%
	5-9 years		−1.179%	−1.259%–−0.982%
	10-19 years		0.110%	0.106%–0.178%
CAKUT	<1 year		−0.863%	−3.410%–1.778%
	<5 years		−1.051%	−3.440%–1.310%
d. **AAPC of CKD DALYs stratified by socio-demographic index (SDI) index.**				
	**SDI**		**AAPC**	**95% CI**
CKD due to Type 1 diabetes	High SDI		−2.634%	−2.653%–−2.594%
	High-middle SDI		−2.960%	−2.992%–−2.941%
	Middle SDI		−2.241%	−2.347%–−2.167%
	Low-middle SDI		−1.306%	−1.476%–−1.256%
	Low SDI		−1.402%	−1.433%–−1.333%
CKD due to Type 2 diabetes	High SDI		−1.712%	−1.837%–−1.678%
	High-middle SDI		−3.875%	−3.948%–−3.713%
	Middle SDI		−2.559%	−2.589%–−2.529%
	Low-middle SDI		−1.212%	−1.224%–−1.158%
	Low SDI		−1.225%	−1.254%–−1.148%
CKD due to Hypertension	High SDI		−1.912%	−2.029%–−1.787%
	High-middle SDI		−2.563%	−2.575%–−2.553%
	Middle SDI		−1.675%	−1.726%–−1.596%
	Low-middle SDI		−1.157%	−1.242%–−0.776%
	Low SDI		−1.345%	−1.372%–−1.335%
CKD due to Glomerulonephritis	High SDI		−0.849%	−0.952%–−0.786%
	High-middle SDI		−1.643%	−1.771%–−1.527%
	Middle SDI		−0.720%	−0.864%–−0.645%
	Low-middle SDI		−0.138%	−0.139%–−0.083%
	Low SDI		−0.086%	−0.168%–−0.017%
CAKUT	High SDI		−2.456%	−4.298%–−0.962%
	High-middle SDI		−3.245%	−5.197%–−1.351%
	Middle SDI		−1.949%	−3.801%–−0.410%
	Low-middle SDI		−1.911%	−4.546%–1.061%
	Low SDI		−1.308%	−4.710%–1.913%
e. **AAPC of CKD DALYs across various regions.**				
	**Region**		**AAPC**	**95% CI**
CKD due to Type 1 diabetes	Asia		−2.314%	−2.396%–−2.263%
	Africa		−1.111%	−1.194%–−0.941%
	America		−1.270%	−1.533%–−1.230%
	Europe		−1.354%	−1.462%–−1.199%
CKD due to Type 2 diabetes	Asia		−2.187%	−2.235%–−2.152%
	Africa		−1.372%	−1.403%–−1.381%
	America		−1.249%	−1.302%–−1.214%
	Europe		−4.642%	−4.919%–−4.291%
CKD due to Hypertension	Asia		−0.810%	−0.920%–−0.786%
	Africa		0.144%	0.115%–0.260%
	America		0.087%	0.079%–0.098%
	Europe		−0.973%	−1.049%–−0.818%
CKD due to Glomerulonephritis	Asia		−1.933%	−1.973%–−1.538%
	Africa		−1.096%	−1.180%–−0.980%
	America		−1.693%	−1.806%–−1.538%
	Europe		−2.431%	−2.466%–−2.425%
CAKUT	Asia		−1.942%	−3.745%–−0.164%
	Africa		−1.291%	−0.484%–3.178%
	America		−1.579%	−3.248%–0.150%
	Europe		−1.525%	−3.079%–−0.017%

**Fig 5:**
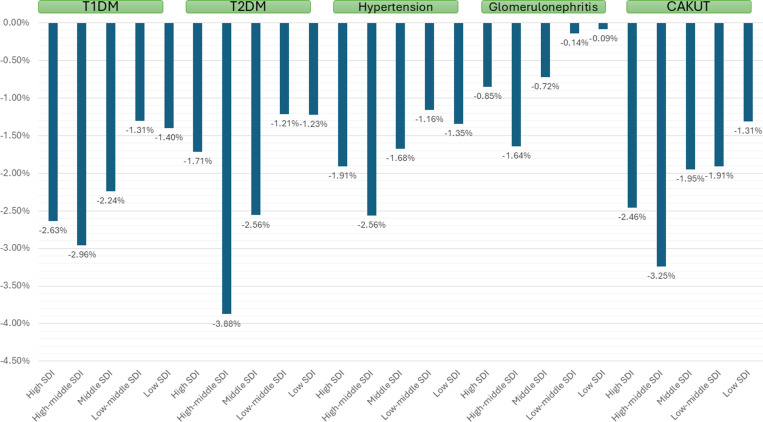
AAPC of CKD DALYs due to different causes between 1990 and 2021 stratified by socio-demographic index (SDI) index.

The decrease in overall DALYs over 1990–2021 was observed to be higher in girls as compared with boys and in younger versus older age (age-wise data was available only for T1DM and glomerulonephritis). With respect to the SDI, the decrease in overall DALY over 1990–2021 was greater among those with higher SDI as compared with lower SDI with a declining trend (Table 4d and Fig 5). With respect to the geography, the decrease in overall DALY over 1990–2021 was observed to be higher in Europe and Asia as compared with America or Africa (Table 4e). High-middle SDI countries and Asia had the highest decrease in death rates due to CAKUT.

## Discussion

The overall incidence, prevalence, mortality, and DALYs of glomerulonephritis, hypertension, T1DM, and T2DM have decreased from 1990 to 2021, with variations based on age, sex, geography, and income level. For each measured etiology, the death rate decreases as age increases. The decrease in pediatric rates among the studied etiologies is complex and multifactorial. However, in our study, older children, particularly those aged 10–19, show higher rates of each etiology. This trend can be attributed to several factors, including advancements in early diagnosis and intervention, improved neonatal and pediatric care, and heightened awareness among healthcare providers about potential kidney diseases in children, and an increased time of chronic illness [4]. Exposures to environmental toxins and pollution, which have been shown to affect renal function adversely, may be factors leading to the progression of CKD in children [16]. The number of children with overweight and obesity, which play a role in the development of CKD, is increasing[17]. Lifestyle factors such as poor diet and physical inactivity also increase the incidence of CKD, whether alone or in combination with obesity and metabolic dysfunction [18,19]. Specifically, adolescents have rising rates of obesity and its sequalae of diabetes, hypertension, and metabolic dysfunction [20–22].

This study also examined sex differences in incidence, prevalence, mortality, and DALYs. Across all four etiologies, boys have a higher incidence and prevalence of the CKD etiologies than girls. This finding aligns with previous studies that suggest biological differences, such as variations in kidney development and function, contribute to this disparity [23,24]. The higher prevalence of CAKUT in boys is also a significant factor [25]. Moreover, genetic predispositions and lifestyle changes, such as increased sedentary behavior and poor dietary habits, contribute to the rising incidence of obesity and metabolic disorders in boys [24]. Our data shows that the AAPC in death rates and DALYs for the CKD etiologies has decreased over time for both boys and girls, with a more significant decrease observed in girls. Girls may have better health-seeking behaviors, adherence to treatment protocols, and potentially less aggressive disease progression compared to boys [23,24,26–28].

Advances in the management and treatment of T1DM, including improved insulin therapies and early detection, have likely contributed to the reduction in mortality rates among children with T1DM [29]. Conversely, the increase in the incidence and prevalence AAPC due to hypertension highlights ongoing challenges in managing this condition in pediatric populations. Hypertension often remains underdiagnosed and undertreated in children, leading to persistent long-term damage and higher mortality rates [30]. The rising prevalence of obesity, which is itself a significant risk factor for CKD, may negate the decreases in incidence and prevalence due to hypertension and diabetes in adolescents.

In our study, the lowest SDI groups had a trend towards the smallest decreases in the incidence and prevalence compared to the middle SDI and high SDI groups, and in the case of hypertension, the largest increases in incidence and prevalence. The low-SDI regions had the least decrease in death rate and DALYs for glomerulonephritis, hypertension, T1DM, and T2DM. While the incidence and prevalence of all five CKD etiologies are relatively lower in the low SDI group, their disease burden is higher. This is likely due to differing risk factor exposures, weak health systems, and limited healthcare resources, highlighting the need for improved planning, tailored interventions, and innovative solutions to address the burden of each of the CKD etiologies in these regions (**Table 5**) [31].

**Table 5:** Key risk factors for CKD in children by continent.

Region	Incidence Rate (per million children)	Prevalence Rate (per million children)	Key Risk Factors
North America	Data not specified	Higher than global average	Diabetes, Obesity
Europe	55-75 pmarp	55-75 pmarp	Diabetes, Hypertension
Latin America (Andean)	Significant increase 1990–2019	Rising prevalence	Low socio-economic status
Latin America (Central)	Significant increase 1990–2019	Rising prevalence	Low socio-economic status
Sub-Saharan Africa	Higher burden due to socio-economic factors	Higher burden due to socio-economic factors	Poor healthcare access, Malnutrition
South Asia	Increasing due to diabetes & hypertension	Increasing due to diabetes & hypertension	Diabetes, Hypertension
East Asia	Data not specified	Data not specified	Urbanization, Pollution
Southeast Asia	Data not specified	Data not specified	Dietary habits, Urbanization
Middle East & North Africa	Increasing trend observed	Increasing trend observed	Hypertension, Obesity

Among the low/middle SDI groups, the incidence and prevalence of glomerulonephritis and hypertension were significantly higher than those diagnosed with T1DM and T2DM. In our study, glomerulonephritis was a significant etiology of death and DALYs. Even in high SDI regions, infectious agents such as hepatitis C and Staphylococcus aureus increased the rates of glomerulonephritis [32]. It is thought that post-streptococcal glomerulonephritis remains the most common infectious agent in low-middle SDI countries, and a growing body of literature suggests it may be a contributor to CKD [33]. The link between glomerulonephritis and CKD requires further research; however, our data suggests that glomerulonephritis may be a key contributor to global CKD burden.

The data shows that the incidence and prevalence of hypertension significantly increased in all global regions relative to T1DM and glomerulonephritis, with the largest increase found in regions with low to middle SDI. Tools for the early identification and treatment of chronic diseases like hypertension in resource limited settings are essential. Community interventions such as HOPE-4 and the WHO HEARTS package has led to greater global awareness for hypertension and more targeted interventions in resource limited settings [34–36]. With metformin being a relatively inexpensive and widely available medication, this may be an effective strategy for low SDI regions. Finally, continued interventions are necessary to decrease the rates of childhood overweight and obesity.

High-middle and high SDI regions had the lowest AAPC in incidence and prevalence with the greatest reductions in death and DALYs across all four etiologies. However, this trend was still homogeneous within different geographical areas. For example, the America region notably had increases in the incidence and prevalence of all four CKD etiologies.

Regarding CAKUT, He et al, in an analysis of GBD 2019, found the highest burden of CAKUT was observed in the 0–4 years age group, with prevalence, mortality, and DALYs reaching 402.28 (299.53–527.67), 1.36 (0.97–1.85), and 134.79 (98.65–178.58) per 100,000, respectively [37]. Global prevalence, mortality, and DALYs decreased, but reductions were slower in low and low-middle SDI regions. Mortality and DALYs were higher in males than females. In 2021, the low and low-middle SDI regions still experience the majority of the CAKUT disease burden.

### Limitations

This study has several limitations. First, the GBD dataset is obtained from hospital records and registries, which likely underreport the true prevalence of the CKD etiologies in the general population and use only a single GFR/albumin measurement for diagnosis. We recognize that differences in healthcare infrastructure, environmental conditions, and access to medical care can also influence the diagnosis and management of CKD and its etiologies. In addition, using a single GFR/albumin level may lead to misclassification of kidney disease. The study did not account for all potential confounding factors, such as environmental exposures and genetic predispositions, which could impact the incidence and progression. In the GBD database, common conditions like posterior urethral valves are included but there is variability in the definition of CAKUT and inclusion of some female genital tract anomalies. Finally, the lack of specific SDI parameters, and lack of data regarding the age group variable make it difficult to generalize some of our findings to specific populations.

## Conclusion

Our study highlights the increasing global burden of pediatric CKD etiologies, including glomerulonephritis, hypertension, T1DM, and T2DM. The data obtained from GBD 2021 underscores the need for improved diagnostic and treatment strategies, particularly in low to middle SDI regions. Targeted interventions addressing the specific etiologies of CKD and promoting early detection and management can help reduce the disease burden and improve health outcomes for children worldwide. Future research should focus on understanding the underlying factors contributing to CKD and developing effective prevention and treatment approaches tailored to different socio-demographic contexts. Additionally, efforts to standardize diagnostic criteria and reporting practices will enhance the comparability of data and facilitate more accurate assessments of CKD prevalence and outcomes. Improving access to healthcare and implementing low-cost screening and treatment programs in resource-limited settings are critical steps toward mitigating the global burden of pediatric CKD.

## Supporting information

S1 File Global burden of disease dataset. (XLSX)
